# Non-contact diagnosis of sleep breathing disorders using infrared optical gas imaging: a prospective observational study

**DOI:** 10.1038/s41598-022-25637-w

**Published:** 2022-12-06

**Authors:** Jun Young An, Hyun Joon Shin, Myunghyun Yang, Do-Yang Park, Jisun Yang, Hyun Jun Kim

**Affiliations:** 1 Department of Otolaryngology, Ajou University School of Medicine, 164 World Cup-ro, Yeoungtong-gu, Suwon, 16499 Republic of Korea; 2 Department of Digital Media, Ajou University, Suwon, Republic of Korea; 3 Sleep Center, Ajou University Hospital, Suwon, Republic of Korea

**Keywords:** Clinical trial design, Medical research

## Abstract

Full-night polysomnography (PSG) is the gold standard for diagnosing obstructive sleep apnea (OSA). However, PSG requires several sensors to be attached to the patient’s body, which can interfere with sleep. Moreover, non-contact devices that utilize impulse radio ultra-wideband radar have limitations as they cannot directly measure respiratory airflow. This study aimed to detect respiratory events through infrared optical gas imaging and verify its feasibility for the diagnosis of OSA. Data collection through PSG and infrared optical gas imaging was simultaneously conducted on 50 volunteers. Respiratory airflow signal was extracted from the infrared optical gas images using an automated algorithm. We compared the respiratory parameters obtained from infrared optical gas imaging with those from PSG. All respiratory events scored from the infrared optical gas imaging were strongly correlated with those identified with standard PSG sensors. Based on a receiver operating characteristic curve, infrared optical gas imaging was deemed appropriate for the diagnosis of OSA. Infrared optical gas imaging accurately detected respiratory events during sleep; therefore, it may be employed as a screening tool for OSA.

## Introduction

Obstructive sleep apnea (OSA) is characterized by recurrent episodes of upper airway obstruction, leading to sleep fragmentation and intermittent hypoxia during sleep^1,2^. Recently, its prevalence has increased due to the aging population and the rising obesity; therefore, the importance of diagnosing this disorder has been emphasized^3^. The gold standard examination for the diagnosis of OSA is attended, in-laboratory, full-night polysomnography (PSG) with multichannel monitoring^4^. However, PSG requires the attachment of several electrodes to the patient’s body to analyze their sleep pattern and is performed in an unfamiliar environment. Thus, patients often feel uncomfortable or sleep poorly, due to which, accurate tests are rarely performed. To overcome these problems, numerous efforts like portable PSG are being made to reduce or eliminate the number of sensors attached to the patient. Recently, non-contact methods for measuring respiratory status during sleep have also been developed. These methods are divided into audio signal and radar. The audio-signal method is used to assess the severity of sleep apnea and snoring through analyzing breathing sound and has advantages in terms of being convenient, economical, and useful as a screening tool^5^. Based on the radar method employed, these devices may either use Doppler radar or impulse radio ultra-wideband (IR-UWB) radar. Doppler radar transmits microwave signals to objects and receives the reflected signals to assess breathing conditions from chest movements^6^. However, the Doppler radar is easily affected by random body movements and has major problems with null-point detection^7,8^. The IR-UWB radar, which has become recently widespread, employs a wide bandwidth and high frequency carrier waves to detect tiny movements with high resolution^9^. Originally developed to control the dimming of streetlights by detecting moving vehicles or people, it has recently been adopted to detect breathing in newborns and premature babies. However, as the IR-UWB determines the breathing state indirectly from the chest movements, the recorded data may differ from the actual breathing state. Moreover, even small movements by the patient may severely disturb the measured signals, such as the respiration rate and the heart rate^9–11^.

In our opinion, infrared optical gas imaging may overcome these limitations. Unlike other approaches, infrared optical gas imaging can directly measure the respiratory airflow, allowing more accurate identification of the breathing and sleep conditions.

Therefore, this study aimed to assess the accuracy and usefulness of infrared optical gas imaging to evaluate breathing conditions during sleep. To verify the same, we compared the PSG data with the corresponding data obtained from infrared optical gas imaging.

## Methods

### Study population and polysomnography

The Otolaryngology Department of Ajou University Hospital selected 50 people between June 2018 and August 2018 through a recruitment notice. These patients provided informed consent for the PSG and for being filmed with infrared cameras during the study. The study was approved by the Clinical Testing Committee of Ajou University Hospital (Approval No. AJIRB-MED-DEV-18-099). All methods were performed in accordance with the relevant guidelines and regulations. The trial registration and Clinical Research Information Service (CRIS) identifier number is KCT0006984. And first registration date is 07/02/2022. The PSG (Embla N7000; ResMed, Amsterdam, the Netherlands) was recorded in sleep laboratory. The PSG data and infrared camera images were recorded simultaneously. Before this study, we assumed that in the infrared optical gas images, the respired air from the nose and mouth may appear distorted due to the oronasal thermistor and the nasal cannula. Thus, the PSG was performed with standard sensors including an oronasal thermistor and a nasal cannula for the first half of the sleep duration; and were removed for the remaining half of the duration. When the oronasal thermistor and nasal cannula were removed, the respiratory events were measured indirectly using chest and abdominal plethysmography belts. Previous studies have demonstrated that assessments of respiratory events with these belts can sufficiently replace measurements by the oronasal thermistor and nasal cannula^12^. The PSG data were manually scored by a sleep specialist, as per the American Academy of Sleep Medicine (AASM) 2012 criteria^13^.

### Infrared optical gas imaging

**Figure 1:**
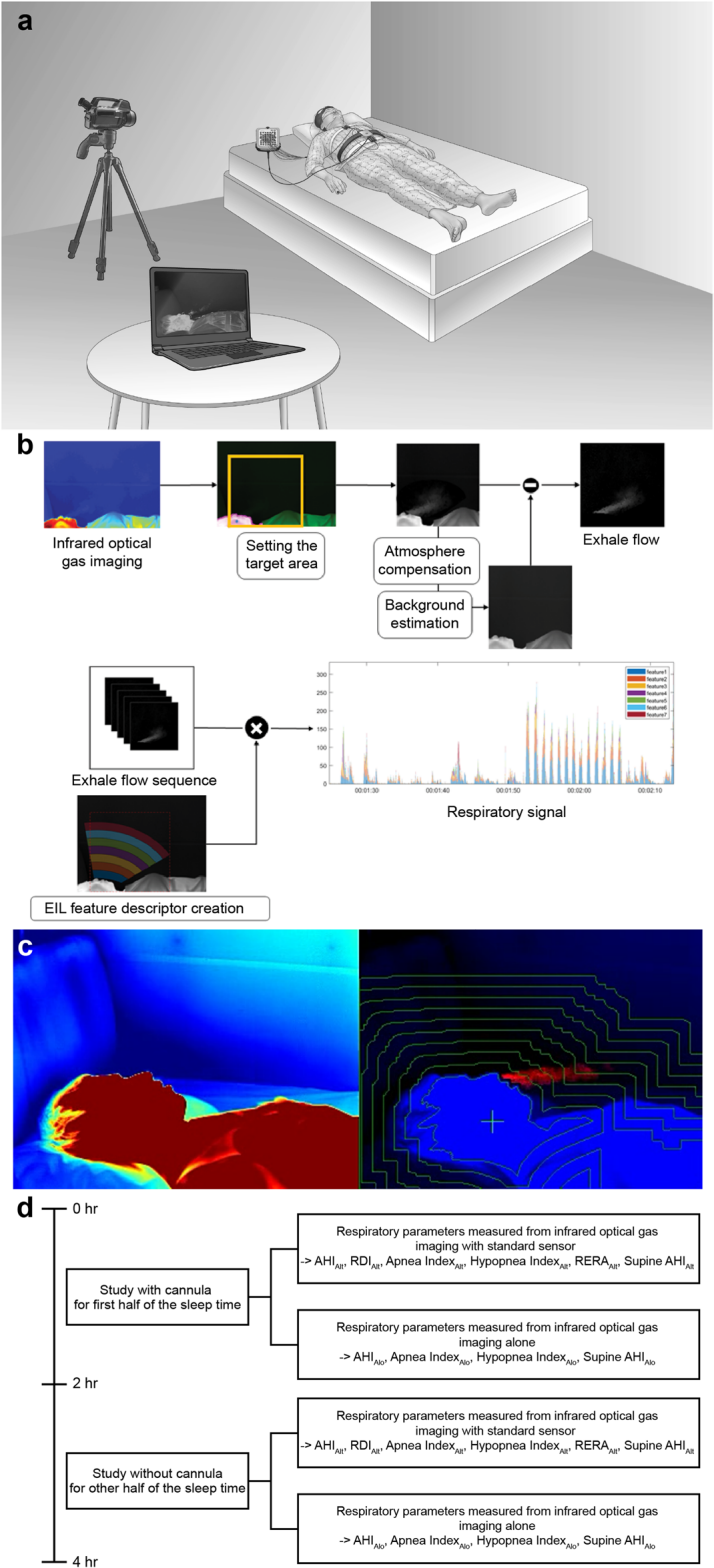
(**a**) Simplified depiction of the experimental setting(created with Adobe illustrator). Infrared optical gas imaging and polysomnography data were obtained simultaneously. The camera was placed 1.6 m from the patient’s face. (**b**) Algorithm for the detection of respiratory events from infrared optical gas images. (**c**) Pre(left) and post(right) images of digital image processing. The video presentation can be viewed online (Additional file 1: Video 1). (**d**) Study design. *AHI* apnea–hypopnea index, *Alo* infrared alone, *Alt* infrared with standard sensor, *EIL* equal-interval layer, *RDI* respiratory disturbance index, *RERA* respiratory effort-related arousal.

Infrared optical gas imaging was performed simultaneously with the PSG using a FLIR GF 343 camera which can detect carbon dioxide (an optical gas camera manufactured by FLIR Systems Ltd, Wilsonville, OR, USA) to capture the carbon dioxide. The camera was positioned 1.6 m horizontally from the patient’s face. To minimize the error rate in the recordings, the camera location was marked on the floor so that all patients were filmed under the same conditions (Fig. 1a). The camera had a resolution of 320 × 240 pixels and contains spectral filter in the spectrum range from 4.2 to 4.4 μm where most carbon dioxide absorb infrared radiation to gain clear gas image. A Noise Equivalent Temperature Difference (NETD) is 15 mK at 30 °C. This value means the minimum temperature difference that can be resolved by the infrared camera. In general, as NETD decreases, the image is better, with 15mK being excellent.

### Measurement of respiratory parameters

The respiratory airflow signal was extracted from the infrared optical gas images using an automated algorithm. The flow chart of this algorithm is schematically represented in Fig. 1b. First, the area where the respiratory airflow was observed was selected as the target region by tracking the face in the infrared image. Then, the background temperature change was measured and subsequently removed from the thermal image, leaving only the temperature change caused by the expiratory airflow. To estimate the distribution and diffusion rate of the exhalation airflow from these separate expiratory airflow images, an equal-interval layer-feature detector was used to convert the respiratory airflow signal^14^. Through digital image processing, we improved the visibility of the airflow image in Fig. 1c. A Video recording of digital image processing can be viewed online (see Additional file 1: Video 1). These respiratory signals replaced the airflow of the oronasal thermistor or nasal cannula (Fig. 1d).

Respiratory parameters such as apnea–hypopnea index (AHI), respiratory disturbance index (RDI), Apnea Index, Hypopnea Index, respiratory effort-related arousal (RERA), and Supine AHI (Infrared with standard sensor [Alt data]; AHIAlt, RDIAlt, Apnea IndexAlt, Hypopnea IndexAlt, RERAAlt, and Supine AHIAlt) were measured from the obtained respiratory airflow signal and standard sensors. The same parameters were measured by only using the respiratory airflow signal without the standard sensors (Infrared alone [Alo data]; AHIAlo, Apnea IndexAlo, Hypopnea IndexAlo, and Supine AHIAlo). Here, Apnea IndexAlo was defined as a > 90% decrease in the average respiratory tract movement for > 10 s, and HypopneaAlo was defined as a > 30% decrease in the average respiratory tract movement for > 10 s, without measuring the level of oxygen saturation.

Infrared optical gas imaging was performed simultaneously with the PSG using a FLIR GF 343 camera which can detect carbon dioxide (an optical gas camera manufactured by FLIR Systems Ltd, Wilsonville, OR, USA) to capture the carbon dioxide. The camera was positioned 1.6 m horizontally from the patient’s face. To minimize the error rate in the recordings, the camera location was marked on the floor so that all patients were filmed under the same conditions (Fig. 1a).

### Statistical analysis

Interclass correlation analyses and Bland–Altman plots were used to confirm the association between respiratory parameters scored from the infrared optical gas images with a standard sensor (Alt data) and from the PSG. The receiver operating characteristic (ROC) curve was used to determine the usefulness of the infrared camera images for sleep apnea diagnosis. Subsequently, respiratory parameters measured using infrared optical gas images alone (Alo data) were compared with the standard data. Finally, a correction analysis was conducted to determine the factors among the demographic data and sleep parameters that caused the differences in the AHI. All statistical analyses were conducted using SPSS version 18.0.0 (SPSS Inc., Chicago, IL, USA). A *P*-value < 0.05 was considered statistically significant.

### Ethics appoval and conent to participate

The Otolaryngology Department of Ajou University Hospital selected 50 people between June 2018 and August 2018 through a recruitment notice. These patients provided informed consent for the PSG and for being filmed with infrared cameras during the study. The study was approved by the Clinical Testing Committee of Ajou University Hospital (Approval No. AJIRB-MED-DEV-18-099).

## Results

The baseline clinical and sleep-related parameters of the patients are summarized in Table 1. The mean AHI was 5.49 ± 9.38 in the polysomnographic measurements.

**Table 1:** Demographic data and sleep parameters of the study population.

Parameter	Value
Age (years)	23.62 ± 6.04
Sex (male)	27 (54%)
Height (m)	1.681 ± 0.093
Weight (kg)	64.63 ± 12.57
BMI (kg/m^2^)	22.75 ± 3.26
Supine position (%)	91.836 ± 20.507
Lateral position (%)	8.164 ± 20.507
Transitions (events)	2.10 ± 4.95
AHI (events/h)	5.49 ± 9.38
RDI (events/h)	11.14 ± 12.87
Total sleep time (min)	107.172 ± 37.707
Sleep latency (min)	20.54 ± 24.81
Sleep efficiency (%)	71.62 ± 24.35
Lowest oxygen saturation (%)	93.31 ± 3.52

Data are shown as the mean ± standard deviation or N (%).

*AHI* apnea–hypopnea index, *BMI* body mass index, *RDI* respiratory disturbance index.

All respiratory parameters scored using infrared optical gas imaging with a standard sensor (Alt data) had a strong positive correlation with the respiratory parameters obtained from the PSG in both experimental conditions, i.e., with and without a cannula (Table 2). Among the measured parameters, the AHI in the two methods were strongly correlated in the presence of a cannula (ρ = 0.868, *P* < 0.001). Similarly, this parameter also showed a strong correlation in the absence of a cannula (ρ = 0.853, *P* < 0.001).

**Table 2:** Interclass correlation between respiratory events scored from infrared optical gas images with standard sensor (Alt data) and from PSG.

	With cannula		Without cannula	
	ρ (95% CI)	*P*-value	ρ (95% CI)	*P*-value
AHI_Alt_	0.868 (0.818, 1.000)	< 0.0001	0.85 (0.797, 1.000)	< 0.0001
RDI_Alt_	0.847 (0.794, 1.000)	< 0.0001	0.853 (0.804, 1.000)	< 0.0001
Apnea Index_Alt_	0.77 (0.717, 1.000)	0.0004	0.788 (0.735, 1.000)	< 0.0001
Hypopnea Index_Alt_	0.894 (0.854, 1.000)	< 0.0001	0.861 (0.816, 1.000)	< 0.0001
RERA_Alt_	0.783 (0.725, 1.000)	< 0.0001	0.825 (0.774, 1.000)	< 0.0001
Supine AHI_Alt_	0.863 (0.813, 1.000)	< 0.0001	0.855 (0.801, 1.000)	< 0.0001

*AHI* apnea–hypopnea index, *Alt* infrared with standard sensor, *CI* confidence interval, *PSG* polysomnography, *RDI* respiratory disturbance index, *RERA* respiratory effort-related arousal.

**Figure 2:**
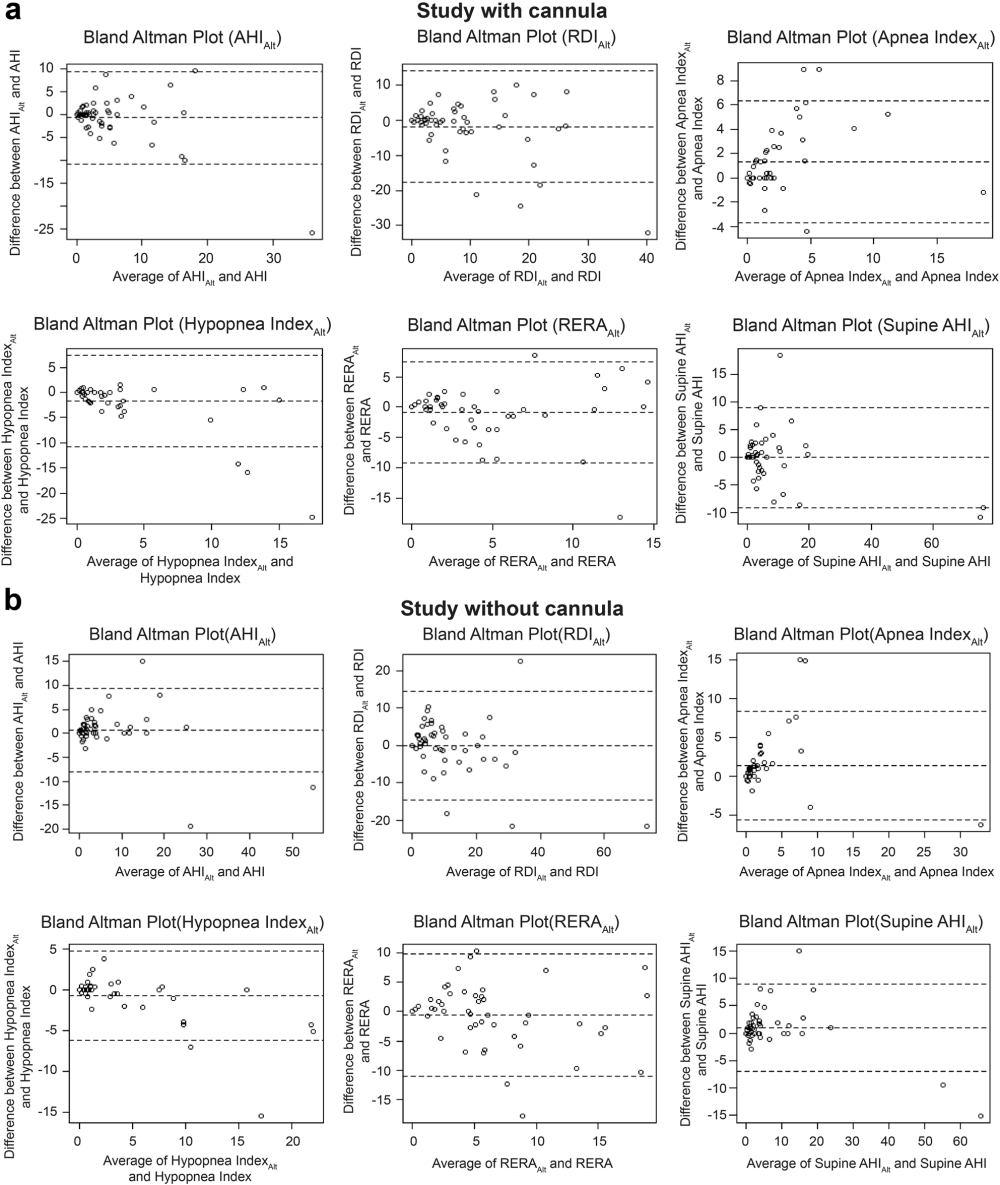
(**a**) Bland–Altman plots for AHI, RDI, Apnea Index, Hypopnea Index, RERA, and Supine AHI in the presence of a cannula when measuring the respiratory parameters with infrared camera and standard sensor. (**b**) Bland–Altman plots for AHI, RDI, Apnea Index, Hypopnea Index, RERA, and Supine AHI under conditions without a cannula when measuring the respiratory parameters with infrared camera and standard sensor. *AHI* apnea–hypopnea index, *Alt* infrared with standard sensor, *RDI* respiratory disturbance index, *RERA* respiratory effort-related arousal.

The Bland–Altman plots of the mean and difference between the polysomnographic data and Alt data are presented in Fig. 2a,b, respectively. All respiratory parameters scored using infrared optical gas imaging with a standard sensor corresponded closely with the parameters obtained from PSG. No significant bias was observed between the measurements of respiratory parameters assessed by the two methods. These results were similar regardless of whether a cannula was present or not.

The same statistical technique was also applied to the respiratory parameters scored by using infrared optical gas imaging alone (Alo data). Again, statistical significance was detected between the Alo data and polysomnographic data (Table 3). The AHI was strongly correlated between the two measurement approaches in the presence (ρ = 0.728, *P* = 0.0236) and absence (ρ = 0.743, *P* = 0.0067) of a cannula. The AHI determined from the Alo data were less correlated than those from the Alt data; however, they were still statistically significant. Moreover, unlike other parameters, the Apnea Index determined from the Alo data had a relatively weak correlation under both conditions, i.e., with and without a cannula (ρ = 0.665, *P* = 0.516 and ρ = 0.687, *P* = 0.253, respectively).

**Table 3:** Interclass correlation between respiratory events scored from infrared optical gas images alone (Alo data) and from PSG.

	With cannula		Without cannula	
	ρ (95% CI)	*P*-value	ρ (95% CI)	*P*-value
AHI_Alo_	0.728 (0.666, 1.000)	0.0236	0.743 (0.688, 1.000)	0.0067
Apnea Index_Alo_	0.665 (0.613, 1.000)	0.516	0.687 (0.630, 1.000)	0.253
Hypopnea Index_Alo_	0.781 (0.729, 1.000)	< 0.0001	0.749 (0.694, 1.000)	0.0036
Supine AHI_Alo_	0.726 (0.663, 1.000)	0.0275	0.740 (0.680, 1.000)	0.0086

*AHI* apnea–hypopnea index, *Alo* infrared alone, *CI* confidence interval, *PSG* polysomnography.

**Figure 3:**
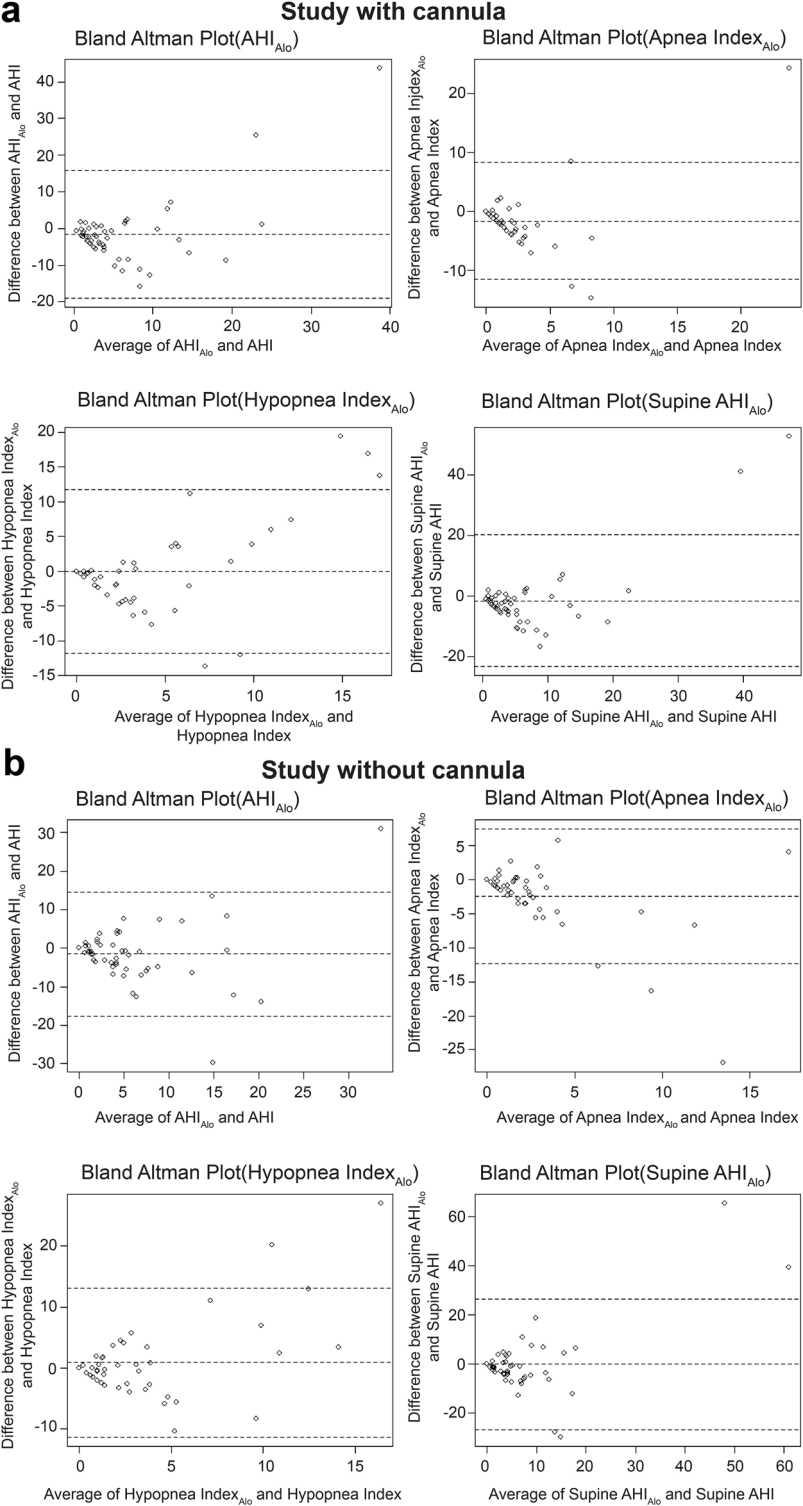
(**a**) Bland–Altman plots for AHI, Apnea Index, Hypopnea Index, and Supine AHI in the presence of a cannula when measuring the respiratory parameters with infrared camera alone. (**b**) Bland–Altman plots for AHI, Apnea Index, Hypopnea Index, and Supine AHI in the absence of a cannula when measuring the respiratory parameters with infrared camera alone. *AHI* apnea–hypopnea index, *Alo* infrared alone.

The Bland–Altman plots of the mean and difference between the polysomnographic data and Alo data are presented in Fig. 3a,b, respectively. Similar to the Alt data, there was no significant difference between the measurements of respiratory parameters obtained from the two methods, and the results were equivalent regardless of whether a cannula was present or not.

**Figure 4:**
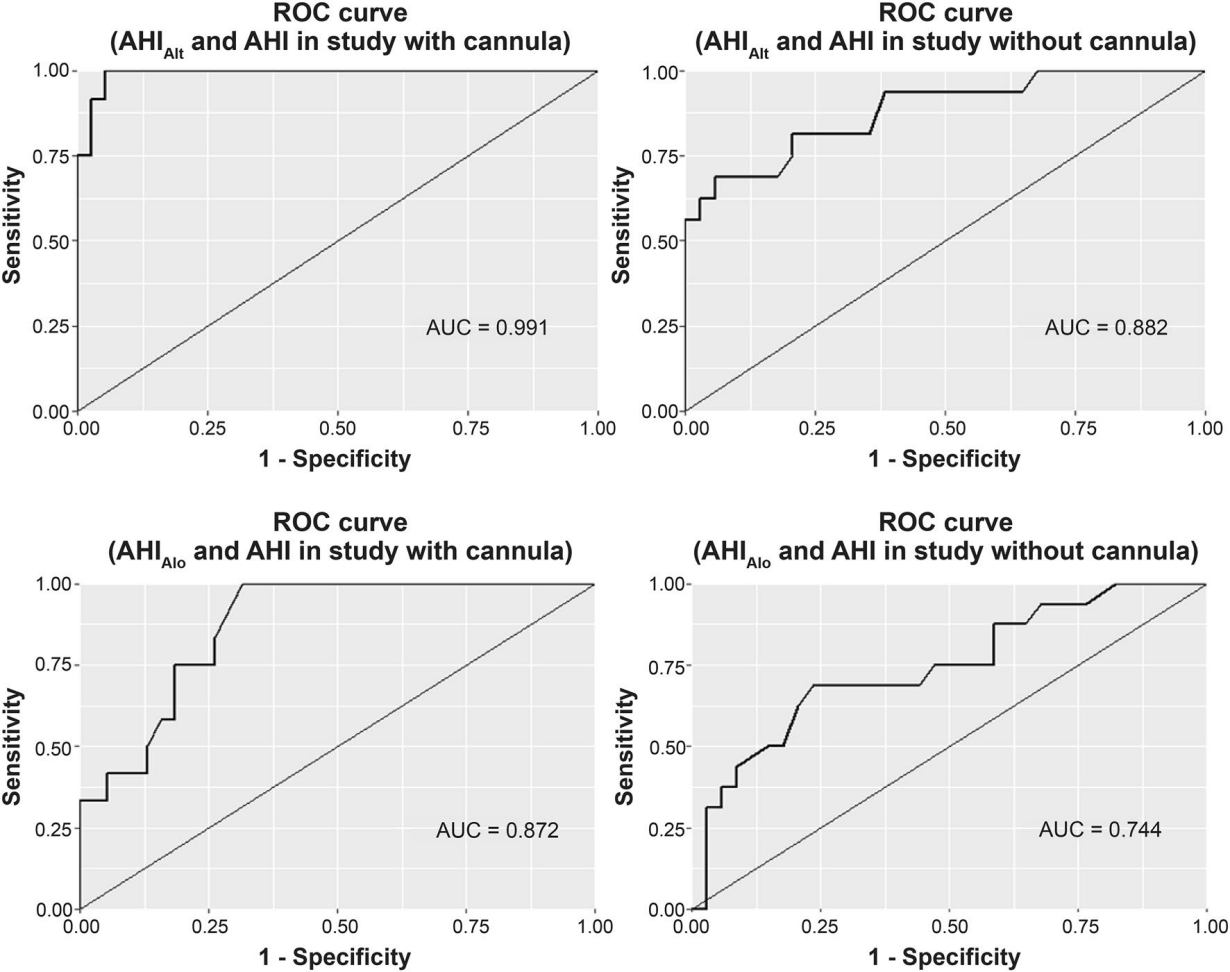
ROC curves to determine the sensitivity and specificity of AHI comparing standard sensor and IR/IR2 sensor data. *AHI* apnea–hypopnea index, *Alo* infrared alone, *Alt* infrared with standard sensor, *AUC* area under the curve, *ROC* receiver operating characteristic.

Figure 4 displays the ROC curve for true- and false-positive rates of sensitivity and specificity of infrared optical gas imaging for OSA diagnosis, based on an AHI cutoff value of five events/h. The area under the ROC curves were more than 0.8 in three groups. Therefore, infrared optical gas imaging may be a valid tool for the diagnosis of OSA.

The demographic and sleep parameters were also analyzed to determine the factors that caused differences between the Alo data and polysomnographic data (Table 4). The Spearman’s rank correlation coefficient analysis showed that the body mass index (BMI) and transition numbers were positively correlated with the differences in AHI between PSG and infrared optical gas imaging in the presence of a cannula. In the absence of a cannula, the BMI, supine position, and lowest oxygen saturation were correlated with the differences in AHI between PSG and infrared optical gas imaging.

**Table 4:** Correlation analysis of AHI differences derived from polysomnographic data versus infrared optical gas imaging data (Alo data).

	With cannula		Without cannula	
	Correlation coefficient	*P*-value	Correlation coefficient	*P*-value
Age	0.1827	0.2040	0.1876	0.1921
Sex	− 0.1111	0.9313	− 0.1817	0.4747
Height	− 0.0574	0.6923	− 0.0137	0.9248
Weight	0.2411	0.0916	0.2178	0.1287
BMI	0.3966	0.0044**	0.3024	0.0328*
Supine	− 0.255	0.0740	− 0.3724	0.0077
Lateral	0.255	0.0740	0.3724	0.0077
Transitions	0.3249	0.0213*	0.2505	0.0793
TST	− 0.163	0.2581	− 0.0852	0.5564
Sleep latency	0.0799	0.5813	0.0084	0.9537
Sleep efficiency	− 0.1086	0.4527*	0.021	0.8849*
Lowest oxygen saturation	− 0.2759	0.0525*	− 0.2852	0.0447*

*AHI* apnea–hypopnea index, *BMI* body mass index, *TST* total sleep time.

*P < 0.05, **P < 0.01.

## Discussion

PSG is the most commonly used tool for diagnosing various sleep disorders^4^. However, there are some limitations to this test. First, PSG requires the attachment of several electrodes to the body to analyze the sleep pattern. Thus, patients often feel uncomfortable or sleep poorly, which often results in inaccurate test results. Moreover, testing in the unfamiliar environment of a hospital can have a ‘first-night effect,’ which increases the waking time and rapid eye movement latency^15^.

To compensate for these problems, a portable sleep monitoring device may be a convenient alternative to use at home; however, simpler and more accurate non-contact devices are being developed. Among these, the most commonly used are wearable devices such as Fitbit, Xiaomi’s Me-Band, and Samsung’s Gear Fit^16,17^, which are mechanical devices that can be worn on the wrist and have actigraphy to measure the user’s movements. If the devices do not move for a defined period of time, the user is considered to be sleeping^18^. Subsequently, deep sleep and light sleep are classified according to the degree of movement, and heart rate variations are analyzed to determine the sleep stage or breathing condition^19^. However, these methods result in inaccurate sleep data. The IR-UWB radar sensors have recently been proposed as a potentially viable tool to monitor heart rhythm and breathing patterns^20^. IR-UWB can detect fine movements of the chest during breathing within a distance of approximately 1 m, enabling contactless respiratory monitoring. Thus, it has been proposed as a tool for diagnosing OSA^8^. However, it has limitations in reflecting the actual breathing conditions, as this method evaluates respiration indirectly through chest movements. Moreover, as the radar detects fine movements, even the slightest movement of the patient or in the surrounding environment may interfere with the measurement of the breathing pattern. Therefore, to overcome these limitations, we used infrared optical gas imaging in our study.

Infrared cameras detect infrared energy and display it as an image. Infrared thermal technologies have recently been used to enable heat detection at entrances of buildings, performance halls, and airports, and are rapidly spreading to newer areas such as forest fire prevention, indoor fire prevention, semiconductor development, and invisible industrial gas detection^21^. The field of infrared optical imaging, which detects only one gas in the air, has also been developed. All gaseous or liquid materials have the characteristic of strongly absorbing infrared rays of their own wavelengths, so only certain gases are detected by infrared optical imaging. By installing a filter in front of the infrared sensor, the camera limits the wavelength band of the measured gas, making the released energy inaccessible to the infrared detector and enabling certain gases to be visually identified^22^.

Carbon dioxide accounts for 4% of the human exhalation airflow, a concentration that is about 100 times higher than that in the atmosphere. Similar to other gases, carbon dioxide also has a unique infrared absorption wavelength of 4.26 µm; therefore, if only carbon dioxide is detected through infrared optical gas imaging, breathing and sleep conditions can be more clearly analyzed.

The results of this study revealed a high correlation between parameters obtained from PSG and those extracted from the infrared gas images. AHI, in particular, showed a strong correlation with polysomnographic measurements regardless of the presence or absence of the cannula (Table 2). Before the study, we hypothesized that the expiratory airflow may be disturbed by the cannula; therefore, a large difference was expected between the data in the presence or absence of a cannula. However, the measured data did not show large differences (Table 2). Moreover, the AHI determined from only the infrared gas images without the information from other sensors was also significantly associated with the AHI obtained from the PSG. This suggests that infrared gas images alone may be a good tool for monitoring respiratory conditions. Furthermore, in the ROC curve analysis based on AHI 5, a diagnostic criterion for OSA, infrared gas images showed high sensitivity and specificity in diagnosing OSA (Fig. 4). Among all conditions, the area under the ROC curve for OSA diagnosis was lowest for the analysis of infrared gas images alone in the absence of a cannula. However, the mean AHI of all study participants was 5.49, indicating that several enrolled participants had no sleep apnea. In future studies, we aim to obtain infrared gas images from patients with severe sleep apnea to determine sleep apnea severity using only infrared gas images.

The average value of most respiratory parameters measured using infrared optical gas images tended to be overestimated than those measured using PSG. The expiratory airflow that could not be captured by an image due to postural changes may be evaluated as apnea or hypopnea. Therefore, sleep specialists should be aware that the AHI may be overestimated by infrared optical gas imaging.

The difference in the AHI values measured from infrared optical gas images and PSG was correlated with the sleep position, BMI, and lowest oxygen saturation. As shown in Table 4, a higher ratio of the supine position during sleep was correlated with increasing similarity of the AHI between the infrared gas image data and polysomnographic data. This is because the patient was supposed to be filmed in the supine position; as long as this position was maintained, the distribution of the respiratory airflow could be accurately measured. If the patient turned to the lateral position, it was difficult to determine the exact AHI value as the lateral view of the patient was not available. In future studies, if several cameras are attached to the ceiling or walls and the breathed air is filmed from various angles, it may be possible to consistently and accurately measure the sleep parameters, regardless of the patient’s posture. Furthermore, the smaller the BMI, the more similar the AHI values were between the infrared gas image data and polysomnographic data. As the AHI was calculated from the respiratory inductive plethysmography signals, there might have been a bias in the AHI measurements of the patients with a high BMI due to reduced plethysmography signal sensitivity, caused by the increased waist and chest circumferences; an assumption that has been confirmed in previous studies^12^. Moreover, the higher the lowest oxygen saturation, the more similar the AHI values were between the two methods.

In recent years, infrared optical cameras have evolved, and more sensitive cameras have been developed for gas detection. Infrared optical cameras are largely divided into thermal detection and quantum detection. The thermal detection method records physical changes in the target according to changes in temperature. As most of these thermal cameras operate at room temperature, they do not need a vacuum for cooling, which makes them low in cost; although the thermal noise in these cameras is higher than that in quantum detection cameras. In contrast, the quantum detection method uses the photoconductive phenomena of semiconductor materials and operates at very low temperatures; therefore, it has low thermal noise and can detect minute changes in the target. However, since the operating temperature is very low, a vacuum must be maintained for cooling, which raises the cost of the camera; therefore, it is mainly used for military purposes^21^. In this study, the quantum detection-type camera GF 343 by FLIR Systems was used for a more accurate measurement of the carbon dioxide concentrations. This type of infrared camera has the disadvantage of being expensive; however, as the technology of infrared sensors develops, the price of quantum detection-type infrared cameras will gradually decrease. Quantum detection cameras operating at room temperature are expected to be widely available soon, making them easier to use at lower prices. This system cannot detect the respiratory efforts, which makes it impossible to distinguish central apnea from obstructive apnea. Hence, the system should not be used for a diagnosis of central apnea.

This study has some limitations. The sleep pattern was only measured for 4 h owing to the small memory capacity of the infrared camera used in this study and the time limit of the cooling system. It is quite short to evaluate sleep status precisely. Therefore, apnea or hypopnea was not measured during the entire duration of sleep. Although total recording time was short to examine overall quality of sleep, it can be enough to assess respiratory status during sleep. In the future, this problem can be solved by increasing the memory capacity or the cooling system. Second, the number of enrolled participants was small, and the study population was mostly limited to a younger-age subset. Further research may produce more clinically relevant results if conducted in a larger number of patients of different ages.

## Conclusions

Infrared optical gas imaging accurately and feasibly detected respiratory events during sleep. The analysis of a patient’s breathing and sleep conditions using infrared optical gas imaging not only has the advantage of being contactless, but also remains unaffected by electromagnetic waves from the surrounding medical devices and other electronics at home. It also has the advantage of efficiently distinguishing the sleep conditions of one person even when two or more people sleep in the same room. With these advantages, the development of a system that analyses sleep conditions using infrared optical gas imaging is an area of great potential in markets directed at sleep healthcare and the diagnosis of respiratory diseases. These devices allow individuals to easily measure their sleep conditions at home, and enable the monitoring of numerous sleep conditions at a low cost in places such as nursing homes and hospitals, where several people are gathered in a room. Moreover, if the breathing disease monitoring system is connected to the network, the patient’s condition could be monitored remotely as well. Therefore, infrared optic gas imaging may be used in various areas of sleep health care.

## Supplementary Information

Supplementary Video 1.

Supplementary Information 1.

## Data Availability

The datasets used and/or analyzed during the current study are available from the corresponding author on reasonable request.

## Abbreviations

AHI: Apnea–hypopnea index
Alo: Infrared alone
Alt: Infrared with standard sensor
AUC: Area under the curve
BMI: Body mass index
CI: Confidence interval
EIL: Equal-interval layer
IR-UWB: Impulse radio ultra-wideband
OSA: Obstructive sleep apnea
PSG: Polysomnography
RDI: Respiratory disturbance index
RERA: Respiratory effort-related arousal
ROC: Receiver operating characteristic
TST: Total sleep time
