# Supplementary material for: Non-contact diagnosis of sleep breathing disorders using infrared optical gas imaging: a prospective observational study
Source: Sci Rep. 2022 Dec 6;12:21052. doi: 10.1038/s41598-022-25637-w (PMC9727032; doi:10.1038/s41598-022-25637-w)
Supplement: Supplementary file 2 — Supplementary Information 1. [file 41598_2022_25637_MOESM2_ESM.pdf]

Description of Additional Supplementary File

File Name: Supplementary Video S1.

Description: Video clips of digital image processing: pre (left) and post (right). The visibility of the airflow was increased on the right video clip compared to the left video clip.
